# Multimodal survival analysis of glioblastoma using whole-slide histopathology, gene expression, clinical variables and language-model-derived mutation features

**DOI:** 10.1038/s41598-026-48666-1

**Published:** 2026-04-21

**Authors:** Tongjie Wang, Javier Alfaro, Paul M. Brennan, Ajitha Rajan

**Affiliations:** 1 School of Informatics, University of Edinburgh, Edinburgh, EH8 9AB United Kingdom; 2 Department for Clinical Neuroscience, NHS Lothian, Royal Infirmary Edinburgh, Edinburgh, United Kingdom; 3 Translational Neurosurgery, Centre for Clinical Brain Sciences, University of Edinburgh, Edinburgh, EH16 4SB United Kingdom; 4 Cancer Research UK Scotland Centre (Edinburgh), Institute of Genetics and Cancer, University of Edinburgh, Edinburgh, EH4 2XU United Kingdom

**Keywords:** Mathematics and computing, Computer science, Cancer, Cancer imaging, Cancer models, Outcomes research

## Abstract

Glioblastoma (GBM) is a highly aggressive brain tumor with poor prognosis, motivating the development of more accurate survival prediction models that can integrate complementary clinical and molecular information. However, existing multimodal survival frameworks often rely on simplified genomic summaries, underuse sequence-context information from mutations, or discard global spatial structure in whole-slide histopathology. In this study, we present MUSA , a multimodal survival framework that integrates three data sources: whole-slide H&E histopathology, tabular clinical and gene expression variables, and language-model-derived mutation features. For histopathology, we use a class-map-based feature extraction pipeline that summarizes slide-level composition, spatial adjacency, and fragmentation patterns. For the molecular branch, we represent missense mutations using protein-sequence-context-based features derived from a language model and evaluate both frozen and survival-supervised representations. These modality-specific features are then fused within a survival prediction framework and evaluated under nested cross-validation. In mutation-only experiments, survival-supervised language-model features outperformed one-hot and VEP-derived baselines. In multimodal benchmark comparisons, MUSA outperformed matched reference models, and in ablation analyses the full trimodal model achieved the best overall median concordance. Together, these results show that mutation-sequence representations and spatial histopathology features provide complementary prognostic information for GBM survival modeling.

## Introduction

Glioblastoma (GBM) is the most common primary malignant tumor of the central nervous system and remains one of the deadliest adult brain cancers despite surgery, radiotherapy, and chemotherapy^1^. Median survival is still limited, which underscores the need for more accurate prognostic models that can support risk stratification and, ultimately, more individualized clinical decision-making. Survival analysis has therefore become an important tool in GBM research, particularly as multiple complementary data types are increasingly available for the same patient.

In routine practice and translational research, GBM prognosis is informed by several distinct sources of information. Histopathological Hematoxylin and Eosin (H&E) slides contain rich morphologic and spatial cues, including nuclear atypia, necrosis, vascular proliferation, and tumor architecture. Clinical variables such as age, sex, treatment-related information, and other patient-level characteristics provide additional prognostic context. At the molecular level, somatic alterations in genes such as *TP53*, *IDH1/2*, and other recurrently altered loci are known to relate to glioma biology and clinical outcome^2–6^. A key challenge is to combine these heterogeneous data sources within a single survival framework without discarding the information that makes each modality clinically useful.

Most existing multimodal survival models still face two important limitations. First, histopathology is commonly reduced to patch-level embeddings extracted from tiled whole-slide images (WSIs), after which patch information is pooled with limited preservation of slide-level spatial organization^7–11^. Although recent foundation and transformer-based WSI methods preserve more context^12^, they may remain difficult to interpret in survival settings and can be computationally demanding. Second, mutation information is often simplified into gene-level indicators or aggregated functional scores, which can miss sequence-context-dependent effects and higher-order interactions among co-occurring variants. In practice, individual variants are frequently analyzed one at a time using carrier-versus-non-carrier survival comparisons, but such strategies do not provide a stable patient-level representation when multiple mutations coexist.

These limitations are especially relevant in GBM. Tumor behavior emerges from interactions across morphology, spatial tissue organization, and molecular alteration patterns rather than from any single modality in isolation. A survival model that uses only tabular summaries, only image patches, or only coarse mutation indicators is therefore likely to underuse available prognostic information. More expressive representations are needed for both histopathology and mutation data, while remaining sufficiently interpretable for biomedical analysis.

In this study, we present MUSA, a multimodal survival framework that integrates three complementary modalities for GBM prognosis: (1) whole-slide H&E histopathology, (2) tabular clinical and gene expression variables, and (3) language-model-derived mutation features. For histopathology, we use an unsupervised class-map-based pipeline to transform WSIs into slide-level spatial descriptors that summarize composition, class-to-class adjacency, and fragmentation patterns. For the molecular branch, we represent somatic missense mutations using protein-sequence-context-based features derived from a language model, and we evaluate both frozen and survival-supervised mutation representations. Clinical variables are incorporated through a tabular branch, and the modality-specific representations are combined in a multimodal survival model.

We evaluate the proposed framework on GBM survival prediction tasks and compare it with established multimodal baselines, including MultiSurv^13^. We further perform unimodal and multimodal ablation analyses to quantify the contribution of each branch. Our results show that mutation-sequence features provide strong prognostic signal, that image-derived spatial descriptors add complementary information, and that the full multimodal framework achieves the best overall performance in our cohort. These findings support the value of combining interpretable histopathology-derived spatial information with language-model-based mutation representations for multimodal survival modeling in glioblastoma.

**Fig. 1:**
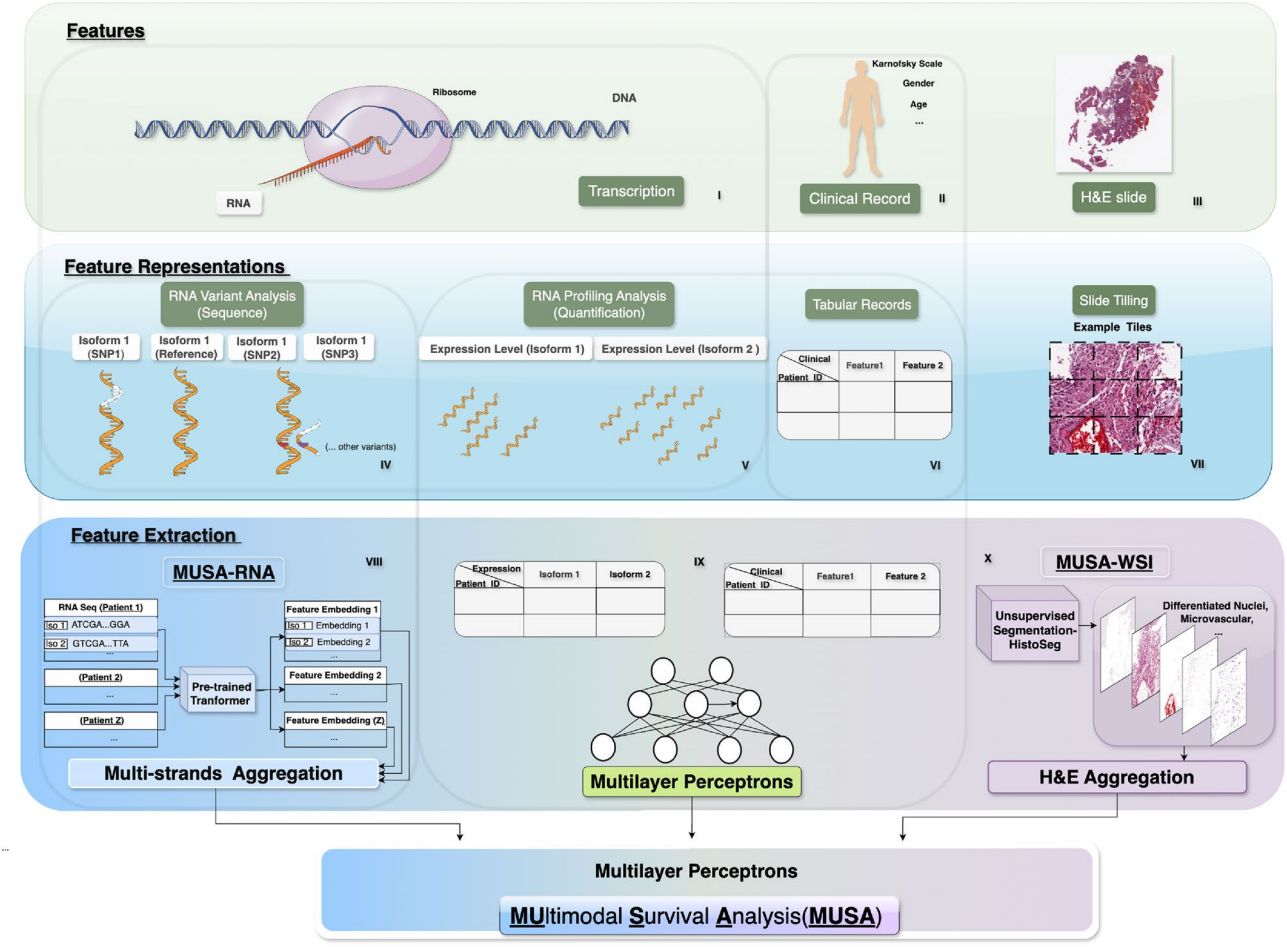
Overview of the study design and multimodal survival framework. The framework integrates three complementary modalities for glioblastoma survival prediction: histopathological H&E whole-slide images, tabular clinical variables, and somatic mutation information. Whole-slide images are processed into class-map-derived spatial descriptors that summarize tissue composition, adjacency, and fragmentation patterns. Somatic missense variants are represented using sequence-context-aware language-model-based features. Clinical variables are encoded through a tabular branch. Modality-specific representations are then fused within the multimodal survival model to generate patient-level risk predictions.

## Results

We evaluated the proposed framework from three perspectives: (1) feature extraction performance on H&E slides, (2) survival prediction based on mutation-derived representations, and (3) multimodal survival prediction using image, mutation, and tabular features. The tabular branch in this study comprises clinical variables and gene expression features.

### Feature extractor assessment for each modality

We first assessed the performance of the modality-specific feature extraction pipelines. For H&E slides, we evaluated the image feature extractor through a nuclei segmentation benchmark and through downstream survival prediction. For the mutation branch, we compared multiple mutation feature representations for survival modeling.

**Fig. 2:**
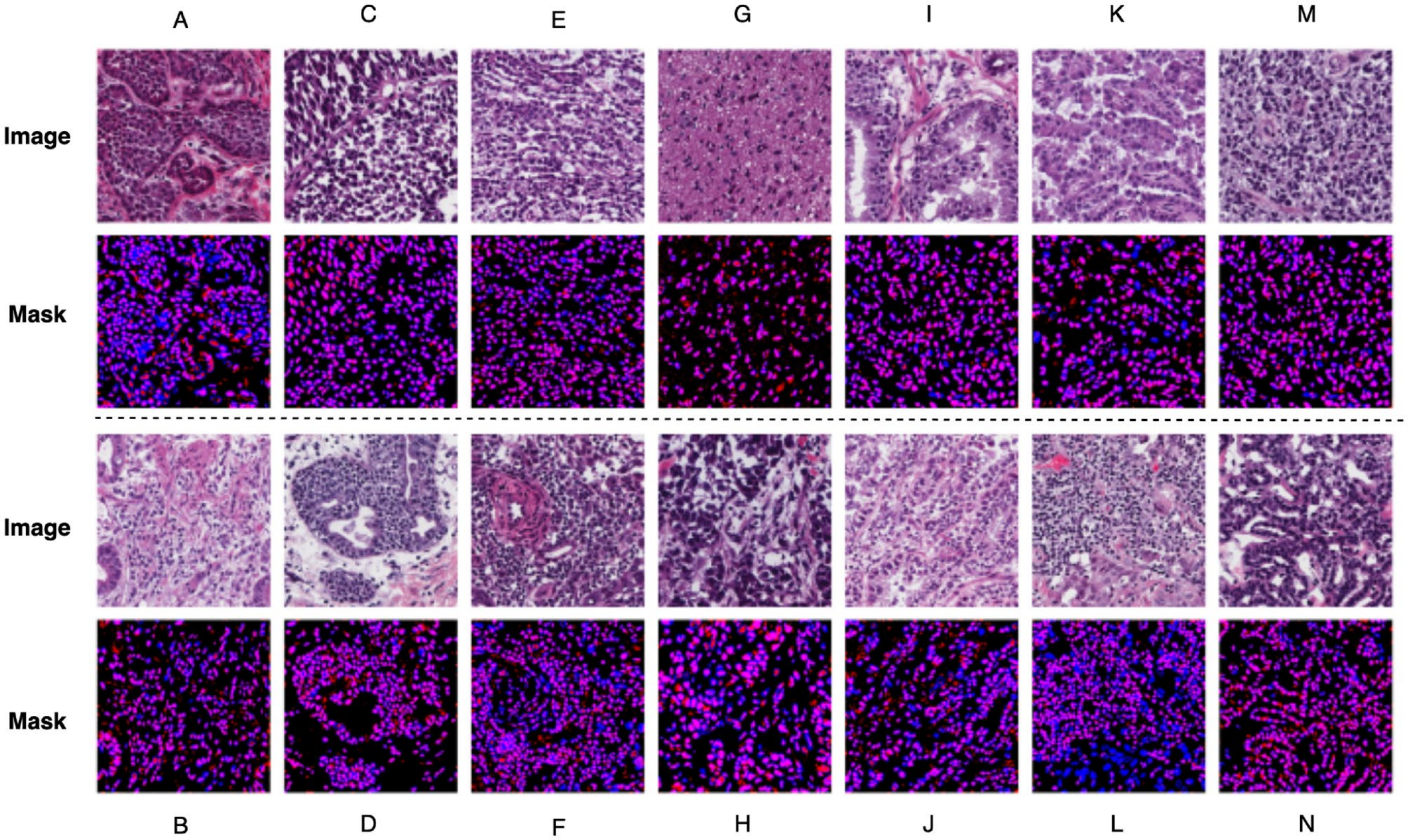
A Comparison Between Annotations (Blue), Nuclei Segmentation Prediction Mask (Red) and Overlap (Purple).

#### Feature extraction assessment on H&E slides

HistoSeg, our unsupervised image segmentation method for H&E slides, is the main component of MUSA-WSI. It is designed to extract base tissue features from whole-slide images, including nuclei, blood vessels, and extracellular matrix or cytoplasmic regions. Because nuclei segmentation is one of the most widely available annotated tasks in digital pathology and has previously been associated with cancer prognosis^14^, we first evaluated the image feature extractor on the nuclei segmentation task and compared it with representative state-of-the-art methods.

We validated segmentation performance on the MoNuSeg multi-organ nucleus segmentation challenge dataset. This dataset contains 37 training images and 14 testing images, with approximately 30,000 manually annotated nuclei. The 37 training images (resolution $1000 \times 1000$ pixels) were used without annotations to self-train our H&E feature extractor. We then applied the trained model to the test set and measured segmentation accuracy using the Aggregated Jaccard Index (AJI) against the reference annotations.

For comparison, we selected representative methods spanning both conventional and deep-learning-based segmentation approaches. These included unsupervised baselines as well as supervised CNN architectures that are commonly used in histopathological image analysis. The reported comparison values were compiled from prior evaluations by Lagree et al.^15^ and Graham et al.^16^, which provide broad benchmarking across segmentation models.

Figure 2 illustrates the visual agreement between the reference annotations and our segmentation output. Blue denotes the ground-truth nuclei mask, red denotes nuclei predicted by HistoSeg, and purple denotes overlap between the two. Most nuclei were captured well by the model, although a small number of images showed lower AJI values. In particular, two test images had AJI scores of 0.57 and 0.58, where some nuclei with less typical morphology or nuclei embedded in more complex surrounding tissue were not cleanly segmented.

Overall, HistoSeg primarily captured the dominant nuclei patterns present in the dataset, which largely consisted of densely stained and morphologically prominent nuclei. Although less frequent or morphologically atypical nuclei were more difficult to recover in some images, the model still achieved strong overall segmentation performance and provided a useful basis for downstream feature extraction from H&E slides.

The primary approach of our segmentation method, HistoSeg, focuses on identifying the most prevalent nuclei type, which, in this dataset, primarily consists of cancer nuclei characterized by dense, dark staining and distinctively large shapes. Less common nuclei types, or those embedded within surrounding tissue structures, are grouped separately. Consequently, some nuclei may not be segmented in certain images. Nonetheless, this approach effectively categorizes dominant nuclei types (we displayed examples of differentiated nuclei and other features in the additional material, Additional Picture 1, using GDC glioblastoma cohort,), providing valuable clinical insights for further refinement in distinguishing additional nuclear variants.

**Table 1:** Comparison of AJI scores across selected models from Lagree et al. and Graham et al.

Unsupervised Models		Supervised Models			
Model	AJI Score	Model	AJI Score	Model	AJI Score
Otsu	0.05	U-Net(VGG-16)	0.49	U-Net(DenseNet-121)	0.47
Watershed	0.08	U-Net(VGG-19)	0.48	U-Net(DenseNet-201)	0.51
Fiji	0.34	U-Net(ResNet-50)	0.49	U-Net(Inception-v3)	0.44
CellProfiler	0.37	U-Net(ResNet-101)	0.47	Mask R-CNN	0.53
QuPath	0.43	U-Net(ResNet-152)	0.44	U-Net Ensemble	0.49
SegNet+WS	0.51	DCAN	0.53	Micro-Net	0.56
SegNet	0.38	DIST	0.56	CIA-Net	0.62
**HistoSeg**	**0.65**	CNN3	0.51	HoVer-Net	0.62

We next applied the image feature extraction pipeline to survival prediction using publicly available whole-slide images from the TCGA glioblastoma cohort.

First, the unsupervised segmentation model was used to transform whole-slide images into quantitative slide-level descriptors for survival analysis. These descriptors summarize spatial and compositional properties derived from the learned class maps and were designed to capture clinically meaningful tissue organization patterns. We then evaluated these features in downstream survival models using the concordance index (C-index) as the primary metric.

Table 2 summarizes the comparison between our image-based survival models and several published alternatives, including Gigapath^12^, DeepGraphSurv^17^, GNC-Cox^18^, and WSISA^19^. In our experiments, MUSA-WSI-COX achieved a C-index of 0.61 and MUSA-WSI achieved a C-index of 0.63, outperforming the baseline CNN model (0.51) as well as the compared reference methods shown in the table, including Gigapath (0.58), DeepGraphSurv (0.59), GNC-Cox (0.53), and WSISA (0.58). These results indicate that the proposed class-map-based image representation captures survival-relevant information from H&E slides while retaining a higher degree of interpretability than black-box tile-embedding approaches.

**Table 2:** Comparison of image-based models for predicting GBM prognosis using the concordance index (C-index).

Model/Method	Whole Slide Based		Tile based	
	CNN	Transformer	CNN	Transformer
Gigapath		0.58	/	/
DeepGraphSurv	/	/	0.59	/
GNC-Cox	/	/	0.53	/
WSISA	/	/	0.58	/
MUSA-WSI-COX	0.61	/	/	/
Baseline CNN	0.51	/	/	/
MUSA-WSI	0.63	/	/	/

### Survival analysis based on language-model feature extraction from genomic mutations

We compared multiple mutation-derived feature representations for survival prediction, including gene-level one-hot encoding, VEP-derived score features, frozen protein language model log-likelihood ratio (LLR)-derived features, and survival-supervised fine-tuned language-model features. Performance was evaluated using the test-set concordance index (C-index), reported as mean ± standard deviation across five repeated splits. Repeated outer splits with inner validation were used for model selection and hyperparameter tuning, and all feature scaling steps were fit on the training data only within each split. The survival-supervised feature extractor was fine-tuned only within the training portion of the nested evaluation pipeline to avoid leakage from the outer test folds.

**Table 3:** Comparison of mutation feature extraction strategies for survival prediction (test C-index, mean ± SD over 5 repeated splits).

Feature representation	Survival model	Test C-index
Fine-tuned LM features	MLP	0.727 ± 0.095
One-hot mutation features	MLP	0.647 ± 0.066
Fine-tuned LM + one-hot + VEP	MLP	0.646 ± 0.076
One-hot mutation features	RSF	0.645 ± 0.108
Frozen LLR-derived features	MLP	0.632 ± 0.113
LLR + one-hot + VEP	MLP	0.585 ± 0.071
VEP-derived score features	RSF	0.531 ± 0.045
VEP-derived score features	MLP	0.492 ± 0.049

Across the evaluated settings, the survival-supervised fine-tuned language-model representation achieved the best predictive performance (MLP: $0.727 \pm 0.095$), exceeding the best one-hot baseline (MLP: $0.647 \pm 0.066$) and all VEP-derived score baselines (best: RSF: $0.531 \pm 0.045$). Even without survival-specific fine-tuning, the frozen LLR-derived representation (MLP: $0.632 \pm 0.113$) also outperformed the VEP-derived score features. These results support the use of sequence-context-aware language-model features for mutation-based survival prediction.

An additional finding is that one-hot mutation encoding outperformed VEP-derived score features in this cohort, despite the fact that VEP scores are intended to reflect functional impact. This suggests that, for the present task and sample size, sparse mutation identity patterns may preserve prognostic information that is not sufficiently retained by the selected score aggregation scheme. Finally, the tested concatenation strategies did not improve performance over the best single representation, which may reflect increased dimensionality and redundancy under a limited sample size.

### Survival prediction benchmark comparisons

We next evaluated the prognostic value of our multimodal feature fusion framework for survival prediction in glioblastoma defined according to WHO 2021 criteria. To ensure a fair comparison of model structure rather than input availability, all benchmarked models were evaluated using the same harmonized preprocessing strategy and the same selected feature sets wherever the corresponding modalities were shared. Model performance was assessed using Harrell’s concordance index (C-index) under cross-validation.

#### Benchmark comparison with established multimodal survival models

Our framework integrates tabular features (clinical variables and gene expression), image-derived features, and mutation-sequence representations within a Cox-based multimodal survival model. We compared it against two representative multimodal survival baselines, MultiSurv^13^ and an implementation adapted from Steyaert et al^20^ with different modalities fusion methods. To focus the comparison on model architecture rather than modality availability, all benchmarked models were evaluated using matched preprocessing and comparable feature sets wherever the relevant modalities overlapped. In addition, because the baseline architectures do not naturally support missing modalities, we disabled masking and gated fusion in this benchmark experiment to ensure a fair structural comparison.

**Fig. 3:**
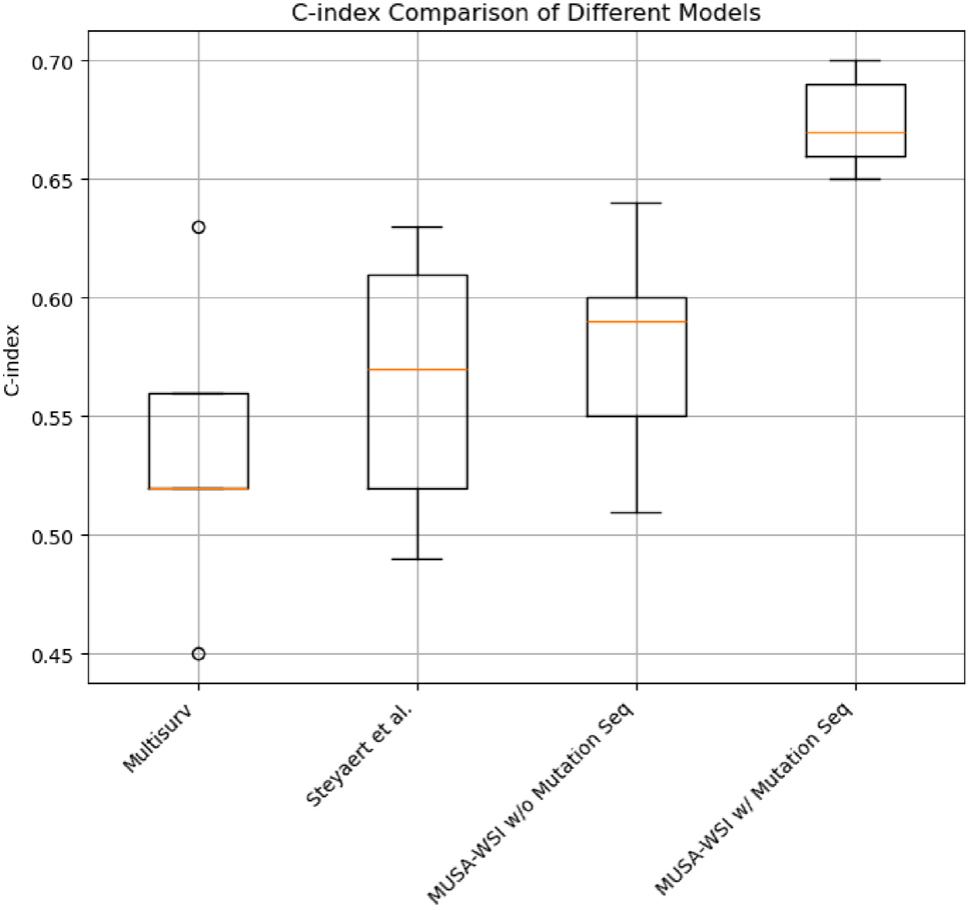
C-index comparison of benchmark multimodal survival models on in-house dataset.

As shown in Fig. 3, the benchmark models followed a clear performance ranking. MultiSurv achieved the lowest performance, with fold-wise C-index values of 0.52, 0.45, 0.56, 0.52, and 0.63 (median 0.52). The model adapted from Steyaert et al. performed better, yielding values of 0.57, 0.61, 0.52, 0.49, and 0.63 (median 0.57). Our model without mutation-sequence features further improved performance to 0.60, 0.51, 0.64, 0.55, and 0.59 (median 0.59). The full model, incorporating mutation-sequence features, achieved the best overall results, with C-index values of 0.72, 0.70, 0.51, 0.59, and 0.62 (median 0.62).

These results indicate that sequence-derived molecular representations provide complementary prognostic information beyond image-derived and tabular inputs. Importantly, because the compared architectures were evaluated on matched feature sets as far as possible, the observed performance differences are more likely to reflect the effectiveness of the model structure and multimodal integration strategy rather than simple differences in input composition. We also observed that enlarging the RNA-related input dimensionality did not further improve performance and could slightly reduce it, suggesting that more compact representations may be preferable in this relatively small cohort.

Overall, the benchmark comparison shows that our framework consistently outperformed the reference multimodal models, and that the inclusion of mutation-sequence features shifted the performance distribution upward despite some fold-to-fold variability. This pattern suggests that molecular sequence information captures survival-relevant signals that are not fully represented by image and tabular modalities alone.

**Fig. 4:**
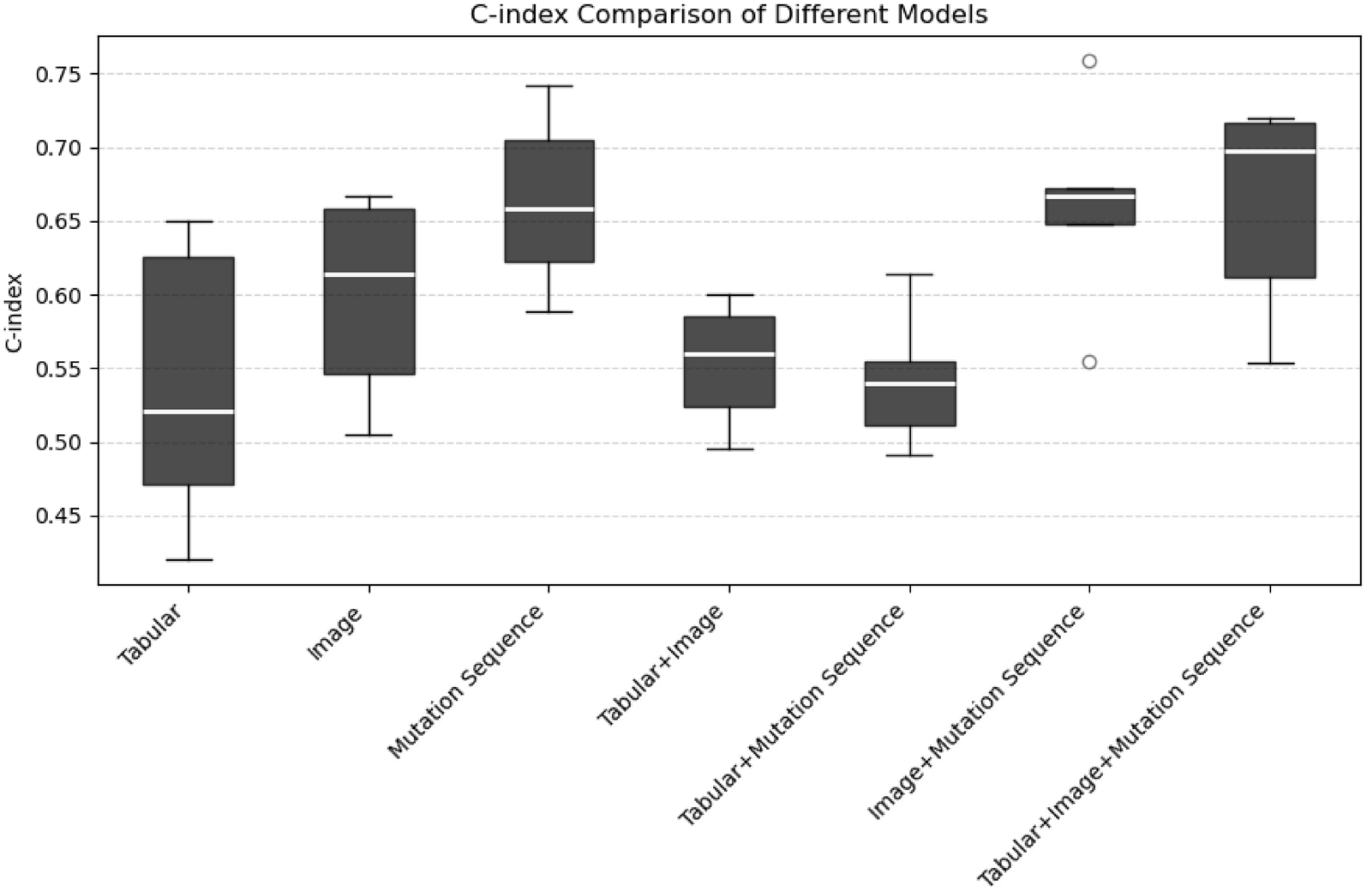
C-index comparison of unimodal, bimodal, and trimodal model variants across cross-validation folds.

#### Internal ablation study of modality contributions

To further dissect the contribution of each data modality, we performed an internal ablation analysis using the same cross-validation framework across unimodal, bimodal, and trimodal model variants. This analysis was designed to determine which modality combinations contributed most strongly to survival ranking performance.

Among the unimodal models, the mutation-sequence branch achieved the strongest overall performance, with fold-wise C-index values of 0.658, 0.622, 0.588, 0.742, and 0.705 (median 0.658). The image-only model showed intermediate performance, with values of 0.667, 0.546, 0.613, 0.658, and 0.505 (median 0.613). The tabular-only model, which used clinical variables together with gene expression features, was the weakest and most variable, with values of 0.650, 0.521, 0.420, 0.625, and 0.471 (median 0.521). These findings suggest that mutation-sequence representations carried the strongest single-modality prognostic signal in this cohort, whereas tabular variables alone were insufficient to provide stable survival discrimination.

The bimodal experiments further clarified the interaction between modalities. Combining tabular and image features resulted in modest performance (0.600, 0.524, 0.496, 0.560, 0.586; median 0.560), while the tabular plus mutation-sequence model remained comparatively weak (0.540, 0.555, 0.512, 0.492, 0.614; median 0.540). In contrast, the combination of image and mutation-sequence features produced the strongest bimodal performance, with C-index values of 0.667, 0.555, 0.672, 0.758, and 0.648 (median 0.667). This indicates that histomorphological and mutation-sequence information are highly complementary for survival prediction.

The full trimodal model, integrating tabular, image, and mutation-sequence features, achieved C-index values of 0.717, 0.697, 0.612, 0.553, and 0.719, corresponding to the highest median performance among all ablation settings (median 0.697). However, its fold-to-fold variation was somewhat broader than that of the image plus mutation-sequence model. Taken together, these results suggest that the survival signal in this dataset is driven primarily by the combination of image and mutation-sequence features, while the tabular branch (clinical variables and gene expression) may provide additional benefit only in a subset of folds.

For the ablation study in Fig. 4, the overall Friedman test was significant ($\chi ^2 = 17.10$, $p = 0.0089$), indicating that modality composition affected survival ranking performance. In planned pairwise Wilcoxon signed-rank tests, the image-plus-mutation model outperformed the image-only model in the expected direction ($p = 0.031$, unadjusted), whereas the contrast between the trimodal model and the image-plus-mutation model was not significant ($p = 0.500$).

In summary, the ablation study supports three conclusions. First, mutation-sequence features were the most informative unimodal predictor. Second, combining image and mutation-sequence features yielded a strong and comparatively stable bimodal model. Third, the full multimodal model achieved the best median performance overall, indicating that integrating all available modalities can further improve survival prediction, although the gain is not uniform across all splits in a limited-size cohort.

## Discussion

Our results support the value of multimodal survival modeling for glioblastoma by integrating image-derived features from H&E slides, mutation-derived molecular representations, and tabular variables including clinical information and gene expression. Across the study, three findings were consistent. First, mutation-derived features provided the strongest unimodal prognostic signal in this cohort. Second, image-derived spatial features contributed complementary prognostic information beyond mutation and tabular inputs. Third, the full multimodal model achieved the best overall median performance in the internal ablation study, although the contribution of each modality was not uniform across all folds. Together, these findings suggest that the prognostic signal in GBM is distributed across multiple biological scales and is better captured by multimodal integration than by any single modality alone. This is the first multimodal implementation to integrate the information from the mutation sequence into survival analysis to the best of our knowledge.

### Feature extractions on H&E slide

Our model’s unsupervised convolutional neural network (CNN) feature extractor for H&E slides demonstrated higher performance in nuclei segmentation tasks, surpassing several state-of-the-art supervised models. Unlike traditional supervised models, which struggle to accurately segment nuclei with atypical or elongated morphologies, our model effectively identifies nuclei with diverse shapes, including elongated and irregular forms. As shown in the example image 5, the supervised model, represented by the GB-U-Net output, fails to capture these nuclei accurately, either missing them entirely (highlighted in red) or introducing extraneous segmentation (in blue). In contrast, our model achieves precise segmentation across a wider morphological range, handling both typical and atypical nuclei with greater robustness. This adaptability to shape variability is critical in clinical settings, where abnormal nuclear shapes often signal pathological conditions. Our results underscore the potential of unsupervised learning approaches in capturing complex and nuanced biological structures, offering a more accurate and reliable tool for histopathological analysis.

**Fig. 5:**
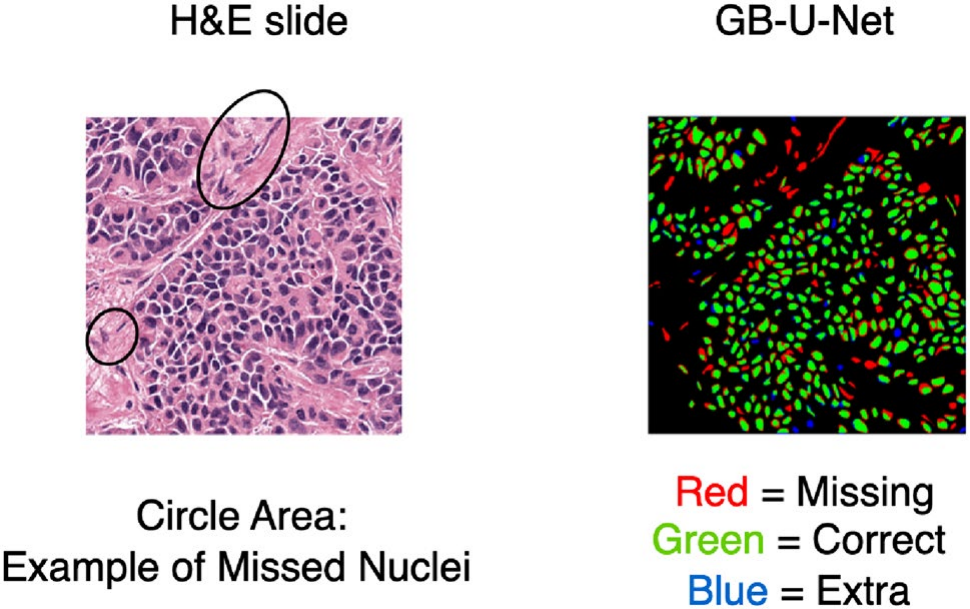
Comparison between H&E slide and the visualized accuracy of a supervised learning model by Lagree et al. on the highest AJI image of the MoNuSeg dataset. Visualized accuracy is color-coded: green indicates true positives, red indicates false negatives, and blue indicates false positives.

### Survival analysis on H&E slide

The survival experiments using H&E-derived features show that slide-level spatial descriptors contain meaningful prognostic information for GBM. Compared with patch-based approaches that summarize isolated tiles, our framework was designed to preserve broader spatial organization within the whole slide. This distinction is important because tissue context can alter the prognostic significance of local morphology: similar-looking regions may carry different biological implications depending on their neighboring structures and their position within the slide, shown in 6.

The competitive performance of the image branch suggests that spatial organization itself is a useful survival signal. Moreover, the use of structured descriptors provides a more interpretable alternative to purely black-box feature embeddings. In a translational setting, this is valuable because clinicians and pathologists are more likely to trust image-based models when the extracted features can be linked back to recognizable aspects of tissue architecture rather than only to latent vectors.

**Fig. 6:**
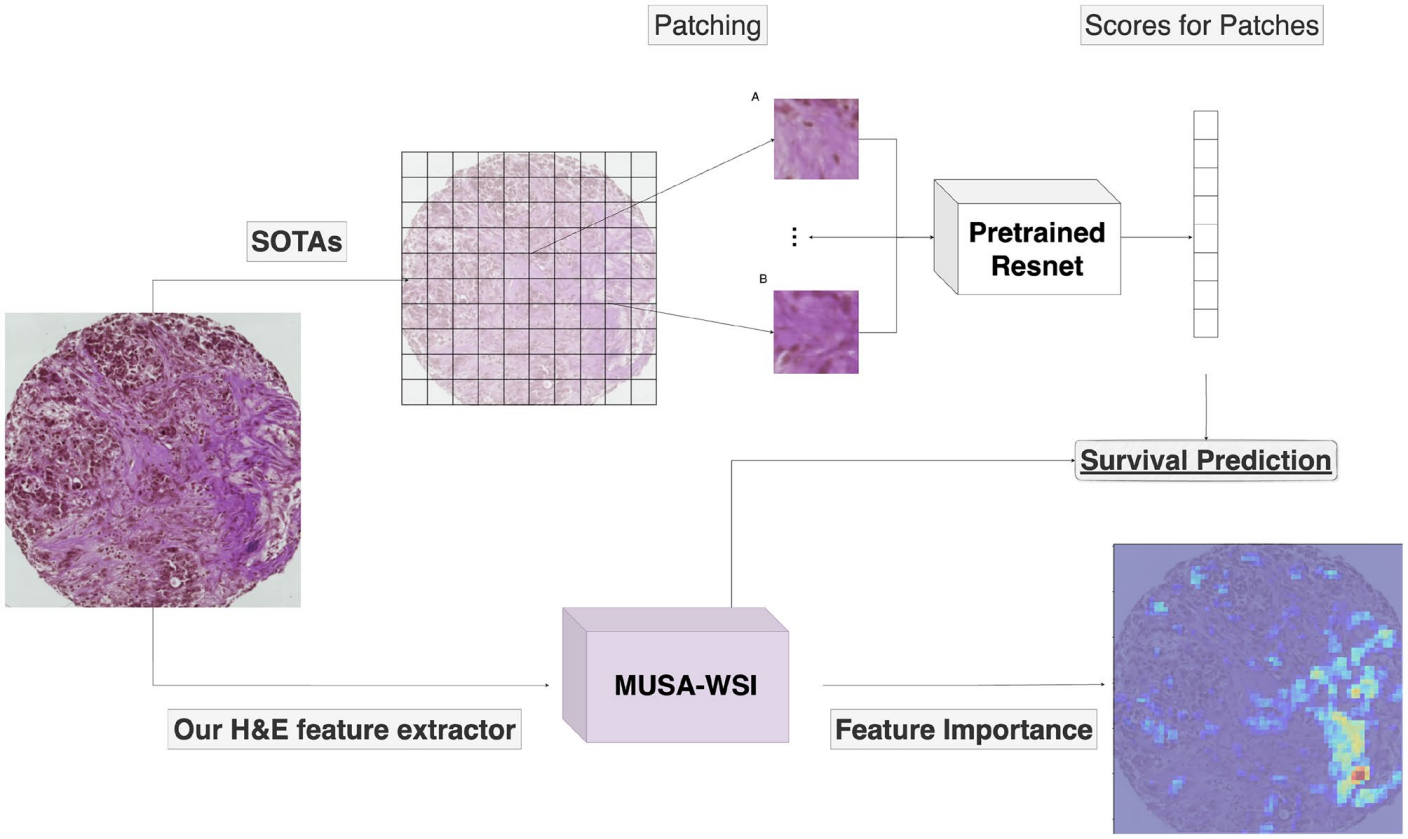
Performance comparison of survival prediction models using our H&E feature extractor vs. SOTA methods relying on patch-based ResNet models.

### Interpretability of class-map-derived survival features and their potential biological meaning

A key advantage of the image branch is that the survival model operates on explicit spatial descriptors derived from the class map rather than on opaque latent embeddings alone. As outlined in Fig. 10, these descriptors summarize class composition, class-to-class adjacency, and within-class fragmentation. This design makes the Cox model directly interpretable: a hazard ratio greater than 1 indicates that a higher value of the corresponding spatial feature is associated with increased hazard, whereas a hazard ratio below 1 indicates an association with reduced hazard. In practical terms, proportion features reflect how much of a learned morphologic state is present, transition and mixing features reflect how different states are spatially interwoven, and fragmentation features reflect whether a class appears as a large contiguous region or as many smaller disconnected islands.

The image CoxPH ranking further suggests that the prognostic signal is driven more by spatial organization than by simple abundance alone. In the current analysis, the strongest image feature was the class-transition term T_1_4 (HR $\approx 0.87$, $p \approx 0.065$), followed by the class-mixing terms mixing_3 and mixing_4 (both HR $\approx 0.91$, $p \approx 0.22$). Although these associations are weaker than the strongest mutation-feature associations and mostly do not cross conventional significance thresholds after penalization, they are directionally consistent with the hypothesis that more compartmentalized or less interdigitated tissue organization may be associated with better outcome, whereas stronger class-class interaction may reflect a more invasive tissue ecology.

The most important point is that these classes should still be interpreted cautiously. Because the class map is learned in an unsupervised manner, class indices such as class 3 or class 4 should not yet be described as definitive biologic entities such as necrosis, stroma, immune infiltrate, or vascular tissue. The safer interpretation is that they represent recurrent learned morphologic states. Accordingly, the image CoxPH plot should be described as identifying which learned states and which interfaces between states are prognostically informative, rather than claiming that a specific histologic compartment has already been validated.

Even with that caution, the image feature analysis is still valuable because it shows that the model is learning not only *how much* of a tissue pattern is present, but also *how it is arranged*. This is also consistent with the qualitative observations in Fig. 6, where morphologically similar patches can contribute differently depending on spatial context. Taken together, the class-map representation and the CoxPH analysis provide an interpretable bridge between whole-slide geometry and patient outcome, while also highlighting the need for future pathology-guided validation of the learned classes.

### Mutation-derived molecular features for survival analysis

**Fig. 7:**
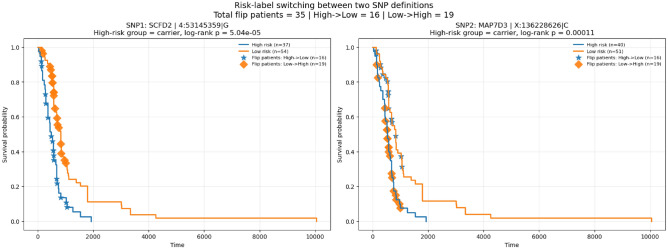
Kaplan–Meier plots showing risk-label switching between two individually significant SNP-based survival definitions. Left: SCFD2 (4:53145359|G), where carriers define the high-risk group. Right: MAP7D3 (X:136228626|C), where carriers also define the high-risk group. Although both SNPs show significant survival separation, patient-level risk assignment is not stable across definitions. A total of 35 patients switched labels between the two SNP-based groupings, including 16 patients changing from high-risk to low-risk and 19 changing from low-risk to high-risk. Star markers denote patients classified as high-risk under the first SNP definition but low-risk under the second, whereas diamond markers denote patients classified as low-risk under the first definition but high-risk under the second. This illustrates the limitation of relying on single-variant definitions for prognostic stratification.

In traditional variant analysis, researchers often evaluate individual variants separately to assess their association with survival outcomes. Although this strategy can identify statistically significant associations, it does not provide a stable patient-level representation when multiple alterations coexist. As shown in Fig. 7, both SNP definitions produced significant Kaplan–Meier separation, with worse survival among carriers of SCFD2 (4:53145359|G; log-rank $p=5.04\times 10^{-5}$) and MAP7D3 (X:136228626|C; log-rank $p=1.1\times 10^{-4}$). However, these two individually significant definitions did not agree at the patient level: 35 patients switched risk labels between the two SNP-based groupings, including 16 reclassified from high-risk to low-risk and 19 in the opposite direction. This illustrates that single-variant survival associations can be statistically significant while still producing unstable and definition-dependent patient stratification.

To address this limitation, we compared increasingly structured mutation representations, from one-hot mutation encoding and VEP-derived score summaries to protein language-model-based features. As summarized in Table 3, the survival-supervised fine-tuned language-model representation achieved the highest predictive performance (MLP: C-index $=0.727\pm 0.095$), outperforming the best one-hot baseline ($0.647\pm 0.066$) and the VEP-derived score baselines (best: RSF $=0.531\pm 0.045$). Even without survival-specific fine-tuning, the frozen LLR-derived representation ($0.632\pm 0.113$) also exceeded the VEP-based models. These results indicate that sequence-context-aware mutation representations retain prognostic information beyond what is captured by sparse mutation indicators or hand-aggregated pathogenicity scores alone.

An additional observation is that one-hot mutation encoding outperformed VEP-derived score features in this cohort despite the biological motivation of VEP-based summaries. This likely reflects a trade-off between aggregation and information loss: compressing variants into a limited set of score summaries may remove prognostic structure that remains available in identity-aware or sequence-aware representations. At the same time, the lack of improvement from naive feature concatenation suggests that simply increasing dimensionality is not sufficient and may worsen the signal-to-noise ratio in a limited cohort.

Our mutation model achieved a test C-index of 0.727 ± 0.095, outperforming the baseline pathological-score aggregation model. Meanwhile, it is also unexpected that one-hot encoder performs better than VEP-derived scores, which later one is more directly correlated with the severity of the mutation. It might also be an indicator that integrating large language model would be a new solution comparing traditional machine learning methods.

### Interpretation of the language-model-based survival predictor

To characterize which mutations contributed to model predictions, we used two complementary interpretation strategies: patient-level attention and remove-one variant ablation, together with feature-level Cox proportional hazards analysis. In the ablation framework, the effect of a mutation was quantified as $\Delta \textrm{risk} = \textrm{risk}_{\textrm{removed}} - \textrm{risk}_{\textrm{full}}$, where $\Delta \textrm{risk} < 0$ indicates that removing the variant decreases predicted risk and is therefore consistent with a risk-increasing contribution, whereas $\Delta \textrm{risk}> 0$ indicates that the removed variant had a risk-decreasing or compensatory contribution.

At the cohort level, the attention analysis highlighted recurrently prioritized genes such as *CLCN6* (81 patients, 83 top-attention hits), *SRSF4* (40 patients, 70 hits), and *HSPG2* (33 patients, 49 hits), indicating that the model repeatedly uses these genes during aggregation. However, attention frequency did not correspond to a single risk direction. As shown in Fig. 9, gene-level ablation identified genes with predominantly positive mean $\Delta$risk, such as *CLCN6*, *HSPG2*, and *STPG1*, as well as genes with predominantly negative mean $\Delta$risk, such as *LAPTM5*, *CROCC*, and *VPS13D*. This indicates that the model is not merely ranking variants by generic deleteriousness, but is instead assigning context-dependent contributions that can differ in direction across genes and patients.

**Fig. 8:**
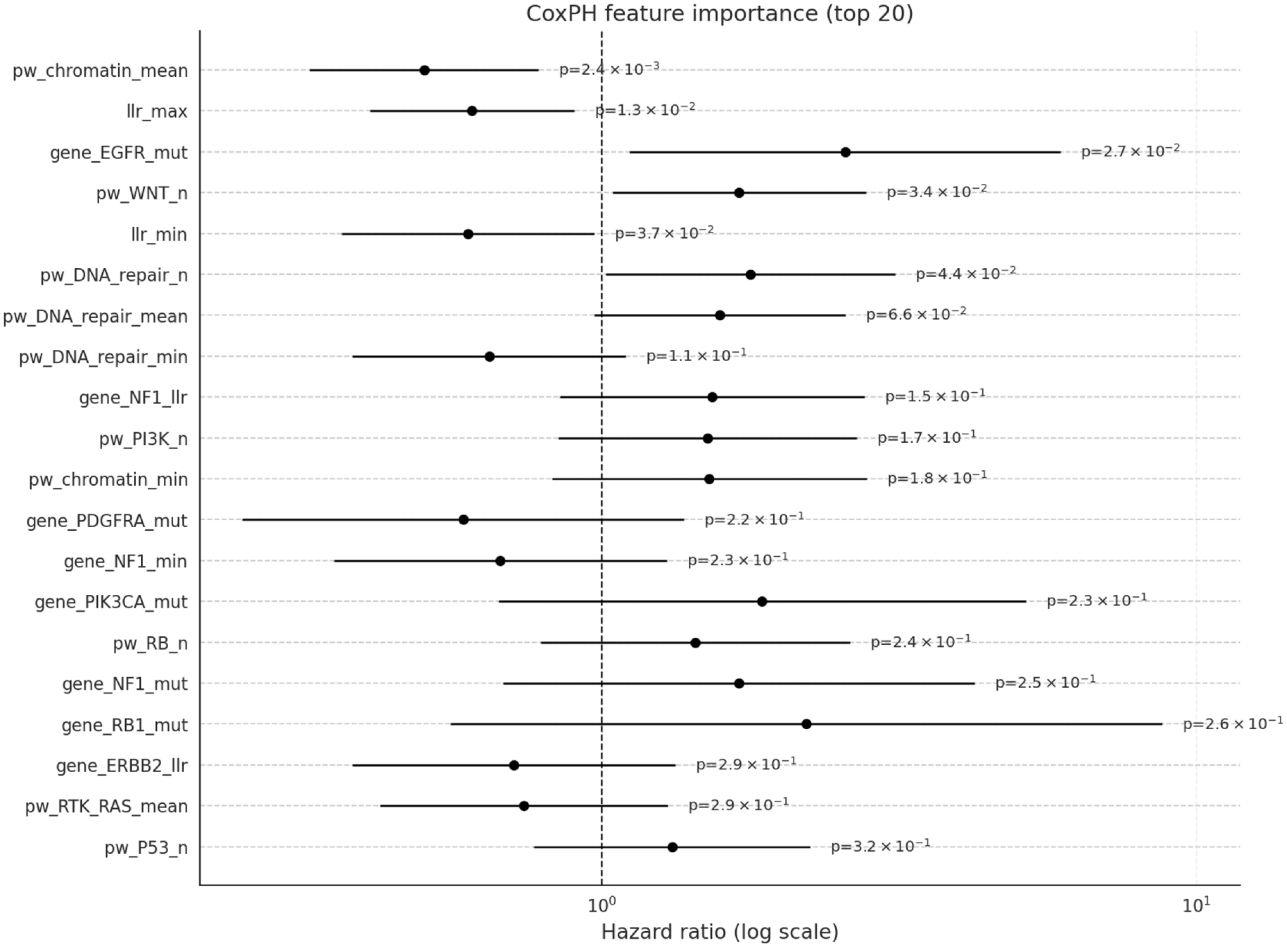
Cox proportional hazards analysis of mutation-derived features. Forest plot of top-ranked mutation-derived features from the Cox model. Points indicate hazard ratios and horizontal lines indicate 95% confidence intervals on a log scale. Features to the right of 1 are associated with increased hazard, whereas features to the left of 1 are associated with reduced hazard. The plotted variables include LLR-based summary statistics, gene-level mutation indicators, and pathway-level burden features.

The CoxPH analysis in Fig. 8 provides a complementary feature-space view of the same phenomenon. The strongest protective-direction association was observed for pw_chromatin_mean ($p = 2.4 \times 10^{-3}$), followed by llr_max ($p = 1.3 \times 10^{-2}$) and llr_min ($p = 3.7 \times 10^{-2}$). In contrast, several biologically structured burden terms were associated with increased hazard, including gene_EGFR_mut ($p = 2.7 \times 10^{-2}$), pw_WNT_n ($p = 3.4 \times 10^{-2}$), and pw_DNA_repair_n ($p = 4.4 \times 10^{-2}$). The significant terms therefore span multiple representation scales, including LLR-distribution summaries, gene-level indicators, and pathway-level burden features.

At the pathway level, the ablation analysis further suggested negative mean $\Delta$risk for PI3K/AKT/mTOR and cytokine/chemokine signaling in the interpreted subset. These findings support the view that the model learns context-dependent rather than uniformly deleterious mutation effects. Nevertheless, the interpretation results should be understood as model-based explanations rather than causal biological conclusions, and should be treated as hypothesis-generating signals for future validation.

**Fig. 9:**
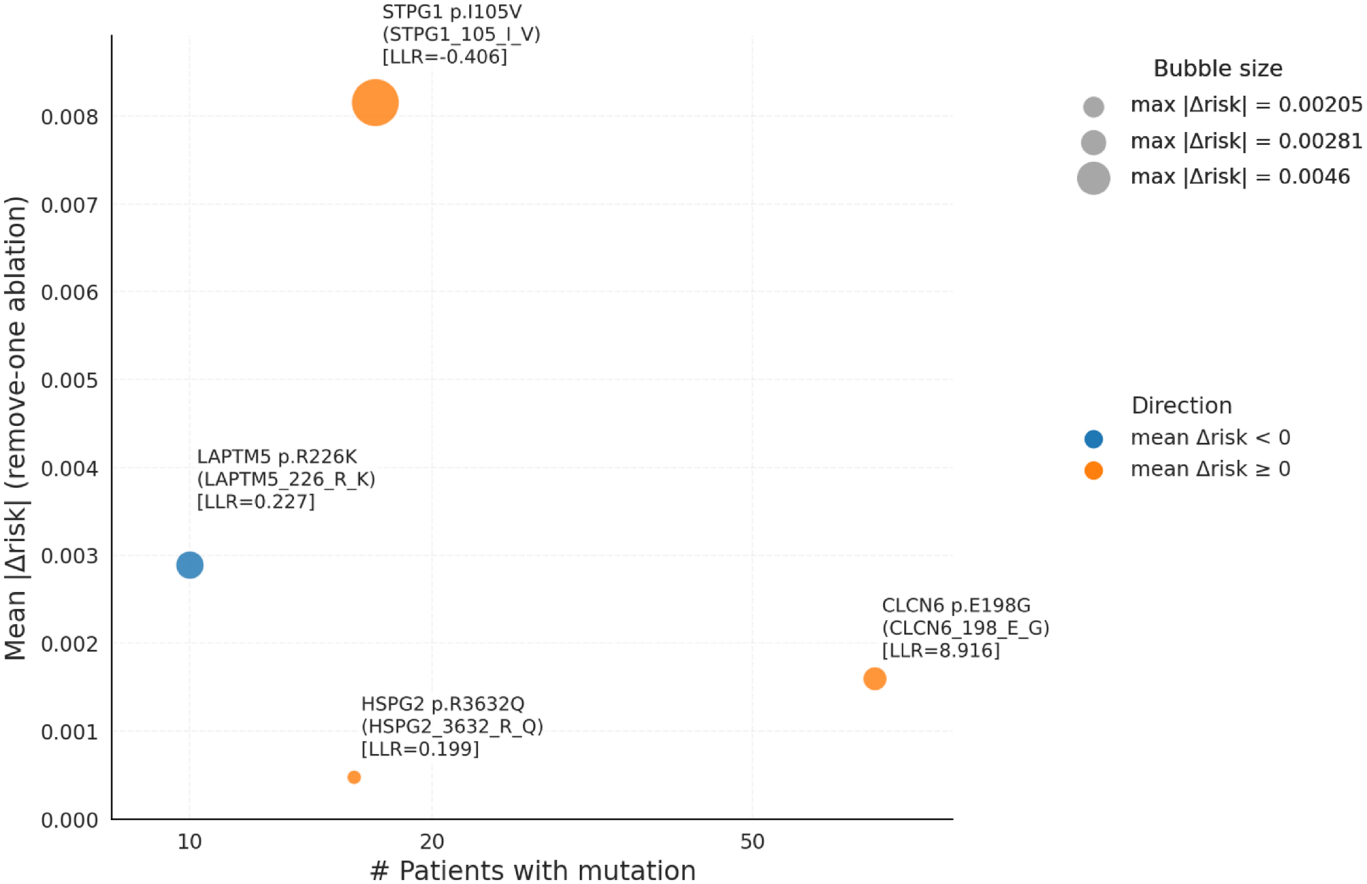
Cohort-level interpretation of the language-model-based mutation predictor. The plot summarizes recurrent mutation signals identified by model interpretation, combining attention frequency and remove-one ablation behavior. Bubble position reflects cohort-level mutation frequency and mean ablation effect, while bubble size reflects the magnitude of the ablation signal. Labeled genes denote recurrently prioritized mutation features with stronger cohort-level influence on predicted risk.

### Multimodal survival analysis and study limitations

The multimodal experiments show that the combination of image and mutation features was particularly strong, indicating that histomorphology and mutation context provide complementary prognostic information. The full trimodal model, which additionally incorporated tabular features from clinical variables and gene expression, achieved the best median performance overall in the internal ablation study. However, this gain was not uniform across folds, suggesting that the benefit of the tabular branch depends on cohort composition, feature quality, and data alignment across modalities.

Several limitations should be considered. First, the cohort size is limited, and all findings are based on internal validation rather than on an external independent cohort. Although nested cross-validation reduces optimism, external validation remains necessary to establish robustness and generalizability. Second, the molecular branch in the current study is focused mainly on missense mutation representations and does not yet incorporate other potentially relevant events such as splice alterations, non-coding variants, copy-number changes, or broader transcriptomic regulation. Third, the image branch prioritizes interpretability through handcrafted spatial descriptors extracted from class maps, but this may limit representational flexibility compared with fully end-to-end learned spatial models. Fourth, multimodal learning in partially aligned clinical datasets remains challenging, and missingness across modalities may affect the stability of fusion performance.

Despite these limitations, the overall pattern of results is encouraging. The study shows that interpretable image-derived spatial features, gene-expression-enhanced tabular variables, and sequence-aware mutation representations can be integrated within a unified survival framework and provide complementary prognostic information. Future work should focus on external validation, extension to broader molecular event types, and improved multimodal fusion strategies under missing-data settings.

## Materials and methods

### Study cohorts and data sources

Three data resources were used in this study. First, the MoNuSeg dataset was used to evaluate the nuclei segmentation performance of the H&E image feature extraction pipeline. MoNuSeg contains high-resolution H&E-stained images with manually annotated nuclear boundaries collected from multiple organs and institutions, and was used here only for segmentation benchmarking.

Second, publicly available whole-slide images and survival data from the TCGA glioblastoma cohort were used for image-based survival analysis. In this part of the study, we evaluated whether slide-level features derived from our class-map-based image representation were associated with prognosis in glioblastoma defined according to WHO 2021 criteria. The TCGA-based survival cohort contained 507 patients after cohort definition and filtering.

Third, our multimodal cohort was used for the integrated survival analyses. This cohort comprised 95 RNA sequencing samples and 135 H&E slides with linked survival information. All patient samples were collected with written informed consent. The study was approved by the East of Scotland Research Ethics Service (EoSRES) as part of the Lothian NRS Bioresource under REC reference 20/ES/0061 (IRAS project ID: 281531). All methods involving human participants, tissue samples, and associated clinical data were carried out in accordance with relevant guidelines and regulations.

In summary, the MoNuSeg dataset was used for nuclei segmentation benchmarking, the TCGA cohort was used for image-based survival analysis, and the in-house cohort was used for multimodal survival modeling.

### Data preprocessing

To prepare each modality for survival modeling, we applied modality-specific preprocessing steps to tabular variables, mutation data, and H&E whole-slide images.

#### Tabular variables: clinical data and gene expression

The tabular branch consisted of structured clinical variables together with gene expression features. Specifically, the clinical covariates were age, sex, diagnosis subtype, disease stage (primary versus recurrence), tumor location, Karnofsky performance score, surgery type, total radiotherapy, and total number of temozolomide (TMZ) cycles. Grade was excluded because it was constant across the cohort (grade 4). Clinical variables were harmonized into a common tabular representation and encoded according to variable type. Continuous variables were standardized, and categorical variables were one-hot encoded within the training data of each cross-validation split.

Gene expression data contained a high-dimensional feature space. To reduce noise and improve tractability, we ranked features using the index of dispersion and retained the highest-ranked features for downstream modeling. The index of dispersion was defined as

1 $$\begin{aligned} I = \frac{\sigma ^2}{\mu } \end{aligned}$$

where *I* is the index of dispersion, $\sigma ^2$ is the variance, and $\mu$ is the mean. This filtering step was performed within the training partition of each split to avoid leakage.

#### Mutation calling and variant curation

To derive mutation features from RNA sequencing data, raw FASTQ files were processed through a standard variant-calling workflow. Reads were aligned to the reference genome using STAR^21^. Picard tools were then used to add read groups and mark duplicate reads. GATK^22^ was used for split-and-trim processing, base quality recalibration, variant calling, and variant filtration. Variants were then standardized into a unified tabular representation containing patient identifier, gene symbol, transcript or protein annotation, amino-acid substitution, and protein position.

For downstream mutation modeling, we retained somatic missense variants with valid amino-acid substitutions and positive protein coordinates. Variant identifiers were defined at the protein level so that each feature represented a specific amino-acid substitution within a gene.

#### Whole-slide image preprocessing

For H&E whole-slide images, slides were divided into tiles and processed using an unsupervised class-map-based image pipeline. The goal of this step was not only to extract local morphologic features, but also to preserve slide-level spatial organization. The segmentation pipeline transformed each tile into a learned class map, and these tile-level maps were then aggregated at the whole-slide level to derive slide-level spatial descriptors for survival modeling.

### Model design

We developed a modality-specific feature extraction pipeline for each data type and then combined the resulting representations in a multimodal survival framework.

#### Unsupervised H&E slide feature extractor

**Fig. 10:**
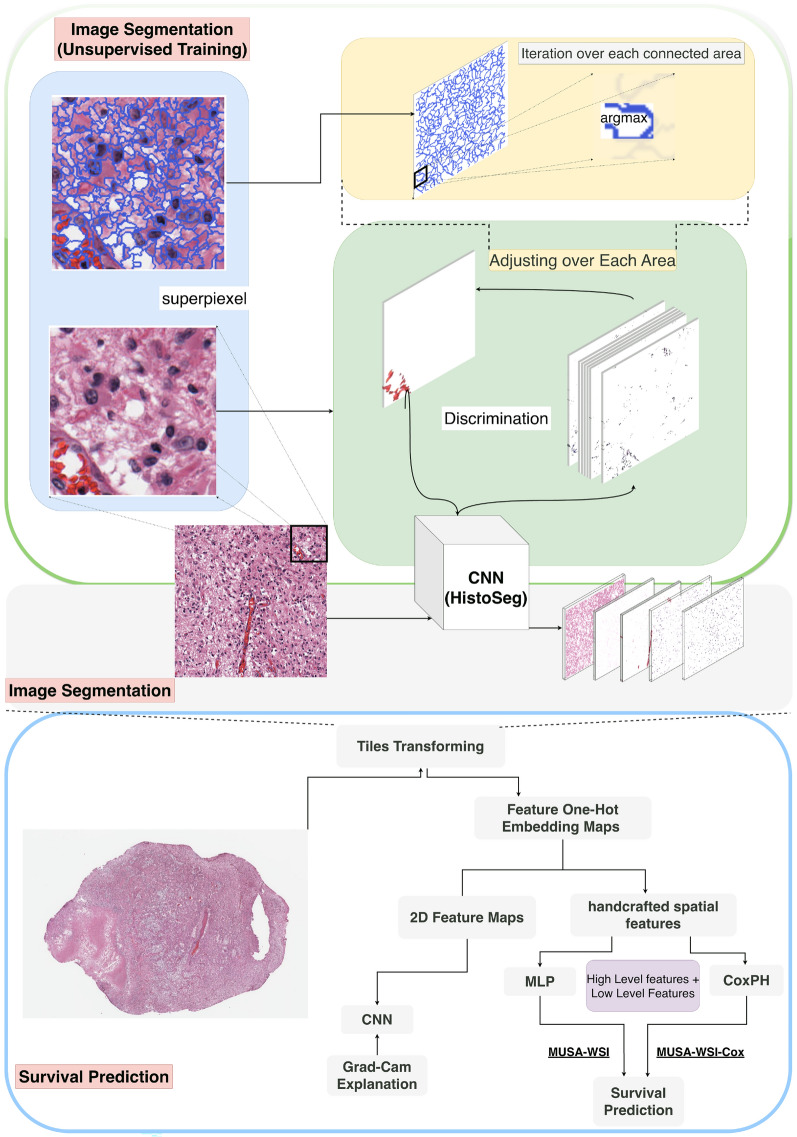
Overview of the WSI Feature Extractor and Survival Prediction Framework. The bottom box presents the survival prediction process, the gray box shows the transformation of segmented tiles into features, and the top box illustrates our unsupervised segmentation method.

The H&E image branch is illustrated in Fig. 10. The preprocessing pipeline begins with an unsupervised image segmentation strategy designed for H&E-stained tissue. A superpixel-based procedure is first used to divide an image tile into locally coherent regions. For each connected region, the dominant feature response across convolutional feature maps is identified by an argmax operation, and this dominant regional label is used to guide unsupervised class discrimination during CNN training. This strategy allows the network to learn recurrent morphologic states without requiring dense manual annotations.

After training, each tile is converted into a discrete class map. These class maps are then aggregated according to their spatial location within the whole-slide image, allowing us to retain global tissue organization rather than treating tiles as isolated inputs. From each class map, we extracted a set of slide-level handcrafted spatial descriptors designed to capture global composition, class-to-class spatial organization, and within-class fragmentation.

First, we computed composition features, including the number of classes, mask height and width, the proportion of pixels assigned to each class, and the entropy of the class distribution as a measure of heterogeneity. Second, we quantified spatial adjacency by counting class transitions along the horizontal and vertical four-neighborhood grid. These counts were normalized to obtain a directed class-transition matrix, and each matrix entry was used as an individual feature. From this matrix, we additionally derived a global heterotypic contact score and per-class mixing scores describing the proportion of boundaries connecting one class to other classes rather than to itself. Third, we quantified fragmentation using connected-component analysis under four-connectivity. For each class, we extracted the number of connected components, the mean component area, and the maximum component area. Together, these descriptors summarize how much of each learned morphologic state is present and how those states are spatially arranged within the slide.

#### Mutation feature extraction using ESM-2

**Fig. 11:**
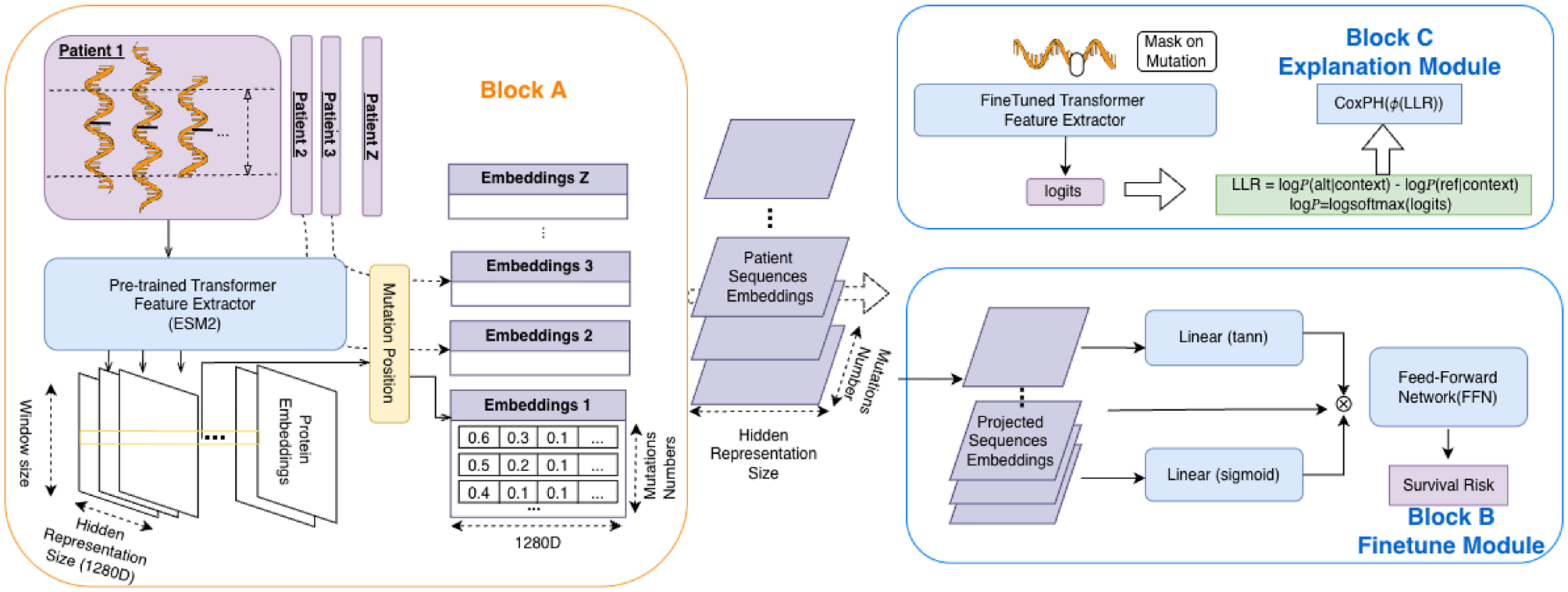
Overview of the mutation feature extraction and multimodal survival framework. Missense variants are represented using sequence-context-aware language-model features and aggregated into patient-level mutation descriptors for downstream survival modeling and multimodal fusion.

The molecular branch is illustrated in Fig. 11. To derive sequence-context-aware mutation features, each curated missense variant was mapped to a reference protein sequence from the Ensembl protein FASTA. Protein sequence resolution followed a hierarchical strategy based on protein identifier, transcript identifier, and gene-level fallback when required. For each variant, a local sequence window centered on the mutated residue was extracted so that the altered amino acid could be interpreted in its surrounding protein context, shown in block A.

Variant effects were first quantified using a frozen ESM-2 protein language model (interpretation analysis was built on finetuned ESM-2 model from Block B, illustrating in the later paragraph). For each sequence window, the residue at the mutation site was replaced with a mask token, and the model was used to estimate the log-probabilities of the reference and alternative amino acids at that position. A log-likelihood ratio (LLR) was then computed as

$$\log P(\textrm{alt}) - \log P(\textrm{ref}),$$

where more negative values indicate that the alternative residue is less compatible with the learned protein sequence distribution.

Because each patient carried a variable number of missense variants, variant-level scores were further aggregated into patient-level features. We summarized the overall distribution of mutation effects using statistics such as mean, minimum, maximum, standard deviation, median, percentile-based summaries, and absolute-effect averages. We additionally quantified the burden of strongly deleterious mutations using threshold-based fractions and counts, and summarized the most disruptive variants using top-*K* features derived from the most negative LLR values.

To preserve biological structure, we also constructed pathway-level and gene-level features. For predefined cancer-related pathways, we calculated pathway-specific mutation counts together with pathway-level mean and minimum LLR values. For selected driver genes, we derived gene-specific mutation indicators and summary statistics. This produced a compact patient-level mutation representation combining sequence-derived functional estimates with biologically structured burden summaries.

In addition to frozen language-model scores, we evaluated survival-supervised mutation representations. In that setting, the mutation feature extractor was fine-tuned only within the training portion of the nested evaluation procedure, so that no information from the outer test folds was used during representation learning. The mutation window is projected to a smaller size of the hidden presentation dimension, then a gated attention mechanism to aggregate all mutations together within each patient, illustrated in Block B.

#### Multimodal data fusion

The final multimodal framework integrates three modality-specific branches: image-derived spatial descriptors from H&E slides, mutation-derived language-model features, and tabular variables comprising clinical information and gene expression. Each modality was first transformed into a branch-specific representation, and these representations were then fused within the survival prediction model.

For the main multimodal experiments, we used a Cox-based fusion framework in which modality-specific embeddings were combined through late fusion. To accommodate partially aligned multimodal data, we implemented masking and gated fusion so that patients with missing modalities could still be used during training. In matched benchmark comparisons with reference multimodal models, masking and gated fusion were disabled when necessary to ensure a fair comparison of model structure under shared input settings.

### Validation strategy

Model development and evaluation were performed using nested cross-validation to reduce selection bias. The outer loop used 5 folds and the inner loop used 3 folds for hyperparameter selection. When both censored and uncensored cases were present, event-stratified splitting was applied; otherwise, standard K-fold partitioning was used.

Within each inner-loop trial, all preprocessing operations, including imputation, one-hot encoding, scaling, feature filtering, and dimensionality reduction where applicable, were fitted exclusively on the training subset and then applied to the validation subset. This prevented information leakage from preprocessing steps. Hyperparameters were optimized by random search over parameters including the dimensionality of selected feature spaces, hidden-layer size, dropout rate, learning rate, and weight decay.

Model performance was quantified on the outer test folds using Harrell’s concordance index (C-index). This validation procedure was used for the TCGA image-based cohort as well as for the multimodal in-house cohort.

## Supplementary Information

Supplementary Information.

## Data Availability

The MoNuSeg dataset used for image segmentation study is available in MoNuSeg challenge repository, https://monuseg.grand-challenge.org/Data/Data used in this work on H&E slide survival analysis are from the GDC Data Portal (https://portal.gdc.cancer.gov/). The patients’ datasets used on multimodal survival analysis are not publicly available due to patients’ privacy concerns of the institutional review board policies on human tissue data.

## Code availability

Code is available from our MUSA Github repository: https://github.com/tongjie-w/MUSA.
