# Supplementary material for: Multimodal survival analysis of glioblastoma using whole-slide histopathology, gene expression, clinical variables and language-model-derived mutation features
Source: Sci Rep. 2026 Apr 21;16:18546. doi: 10.1038/s41598-026-48666-1 (PMC13269476; doi:10.1038/s41598-026-48666-1)
Supplement: Supplementary file 1 — Supplementary Information. [file 41598_2026_48666_MOESM1_ESM.pdf]

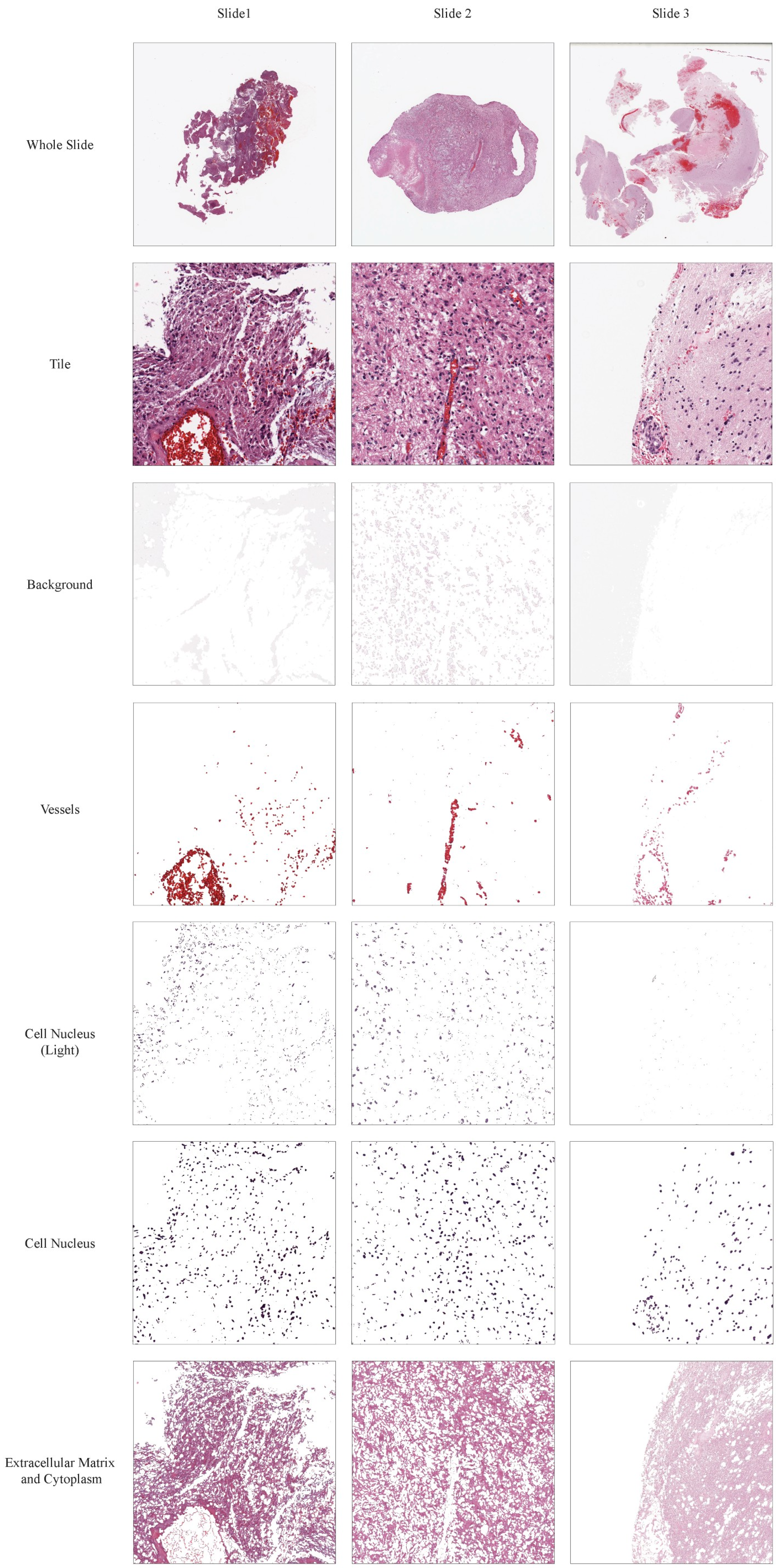
Slide1
Slide 2
Slide 3
Whole Slide
Tile
Background
Vessels
Cell Nucleus (Light)
Cell Nucleus
Extracellular Matrix and Cytoplasm
